# Selective hepatitis B and D virus entry inhibitors from the group of pentacyclic lupane-type betulin-derived triterpenoids

**DOI:** 10.1038/s41598-020-78618-2

**Published:** 2020-12-10

**Authors:** Michael Kirstgen, Kira Alessandra Alicia Theresa Lowjaga, Simon Franz Müller, Nora Goldmann, Felix Lehmann, Sami Alakurtti, Jari Yli-Kauhaluoma, Dieter Glebe, Joachim Geyer

**Affiliations:** 1 Biomedical Research Center Seltersberg (BFS), Institute of Pharmacology and Toxicology, Faculty of Veterinary Medicine, Justus Liebig University Giessen, Schubertstr. 81, 35392 Giessen, Germany; 2 National Reference Center for Hepatitis B Viruses and D Viruses, Institute of Medical Virology, Justus Liebig University Giessen, 35392 Giessen, Germany; 3 Drug Research Program, Division of Pharmaceutical Chemistry and Technology, Faculty of Pharmacy, University of Helsinki, Viikinkaari 5 E, P.O. Box 56, 00014 Helsinki, Finland; 4 VTT Technical Research Centre of Finland, Biologinkuja 7, P.O. Box 1000, 02044 Espoo, Finland

**Keywords:** Viral hepatitis, Drug development

## Abstract

Current treatment options against hepatitis B and D virus (HBV/HDV) infections have only limited curative effects. Identification of Na^+^/taurocholate co-transporting polypeptide (NTCP) as the high-affinity hepatic receptor for both viruses in 2012 enables target-based development of HBV/HDV cell-entry inhibitors. Many studies already identified appropriate NTCP inhibitors. However, most of them interfere with NTCP’s physiological function as a hepatic bile acid transporter. To overcome this drawback, the present study aimed to find compounds that specifically block HBV/HDV binding to NTCP without affecting its transporter function. A novel assay was conceptualized to screen for both in parallel; virus binding to NTCP (measured via binding of a preS1-derived peptide of the large HBV/HDV envelope protein) and bile acid transport via NTCP. Hits were subsequently validated by in vitro HDV infection studies using NTCP-HepG2 cells. Derivatives of the birch-derived pentacyclic lupane-type triterpenoid betulin revealed clear NTCP inhibitory potency and selectivity for the virus receptor function of NTCP. Best performing compounds in both aspects were **2**, **6**, **19**, and **25**. In conclusion, betulin derivatives show clear structure–activity relationships for potent and selective inhibition of the HBV/HDV virus receptor function of NTCP without tackling its physiological bile acid transport function and therefore are promising drug candidates.

## Introduction

Infections with the Hepatitis B (HBV) and D (HDV) viruses are the main cause of hepatocellular carcinoma (HCC) and liver cirrhosis as consequences of chronic hepatitis. Although vaccination is possible, more than 250 million people worldwide suffer from chronic hepatitis based on HBV/HDV infections, and 887,000 deaths can be traced back to this every year^1^. The genome of the enveloped DNA virus HBV consists of 3.2 kb which encode for three envelope proteins, referred to as small (SHBs), middle (MHBs), and large (LHBs)^2^. The 2–48 N-terminal amino acids of the myristoylated preS1 domain (myr-preS1_2-48_ lipopeptide) of the LHBs are essential for virus binding to hepatocytes^3,4^. As HDV is a satellite virus of HBV and bears identical envelope proteins, it also shares this myr-preS1_2-48_ lipopeptide^5^. About 5% of all chronic HBV carriers are additionally infected with HDV^1^. This co-infection causes more rapid disease progression, increased mortality rates, and increased incidence of HCC and liver cirrhosis^6^. To date, there are only few treatment options for HBV and HDV infections available. Chronic hepatitis can usually be controlled by antiviral therapy with nucleoside reverse transcriptase inhibitors and interferon. This is, however, rarely curative. Furthermore, interferon therapy is highly prone to adverse drug reactions and nucleoside reverse transcriptase inhibitors have to be given life-long^7,8^.

A promising novel drug target to block HBV/HDV virus entry into hepatocytes is represented by the Na^+^/taurocholate co-transporting polypeptide NTCP (gene symbol *SLC10A1*), which has been identified as the bona fide hepatic receptor for HBV/HDV^9,10^. It is generally accepted that HBV/HDV entry into hepatocytes essentially involves attachment of the virus particles to heparan sulfate proteoglycans^11^, followed by high-affinity binding of the myr-preS1 domain of the large envelope protein to certain domains of NTCP that finally triggers subsequent steps such as endocytosis of the virus-receptor complex^12^.

It is known for long that an artificial myr-preS1_2-48_ lipopeptide itself can block in vitro HBV/HDV infection in a dose-dependent manner with inhibitory constants (IC_50_) in the low nanomolar range^4^. Based on this, a synthetic analogue of the myr-preS1_2-48_ lipopeptide, called bulevirtide (generic name, formerly known as Myrcludex B), was developed as a therapeutic agent against HBV and HDV infections. Several clinical studies have been successfully completed and very recently, bulevirtide was approved as a new drug to treat hepatitis D in Europe and Russia and will be marketed under the brand name Hepcludex^13^. Furthermore, it was demonstrated that viral DNA (HBV) and RNA (HDV) levels can be reduced significantly in chronically infected patients with a combination of bulevirtide and pegylated interferon alpha (PEG-IFNα)^14^. These data clearly indicate that NTCP is an appropriate drug target to control hepatic HBV/HDV levels. In addition, an HBV/HDV entry inhibitor addressing NTCP might be beneficial to prevent new infections of hepatocytes during chronic infection with HBV/HDV^12^.

Physiologically, NTCP is expressed dominantly at the basolateral membrane of hepatocytes and acts as a sodium-dependent uptake carrier of bile salts to enable their enterohepatic recirculation^15–17^. Thus, NTCP has a dual function as bile salt transporter and virus receptor. It is already known that both functions interfere with each other. The myr-preS1_2-48_ lipopeptide can block the bile salt transport in primary human hepatocytes and NTCP-overexpressing HepG2 hepatoma cells in a concentration-dependent manner. Vice versa, bile salts can prevent myr-preS1_2-48_ lipopeptide binding to NTCP as well as in vitro HBV infection^10,18^. Based on this, it was expected that in clinical trials using bulevirtide as a therapeutic agent, the total plasma bile salt levels would increase significantly. In a current study on healthy volunteers who received 10 mg bulevirtide, which is above the therapeutic dose, total plasma bile salt levels indeed increased 19.2-fold, and single bile salts even higher (e.g. 124-fold in the case of taurocholic acid, further referred to as TC)^19^. This data can be interpreted as dysregulation of the bile acid homeostasis under bulevirtide treatment.

On the basis of the therapeutically used effects of bulevirtide, a promising second strategy for the development of HBV/HDV entry inhibitors targeting NTCP would be to (I) investigate small molecules with oral bioavailability which (II) block virus binding to NTCP, but (III) without tackling its physiologically important bile salt transporter activity at relevant therapeutic drug concentrations. Currently, several studies identified novel chemical entry inhibitors for HBV and HDV by screening for bile salt transport inhibitors^20^ or by screening for inhibitors of myr-preS1_2-48_ lipopeptide attachment and/or in vitro HBV/HDV infection^21^ in appropriate cell culture models overexpressing NTCP. However, none of these studies addressed and fulfilled all of the criteria outlined above. In contrast, the present study aimed to find small molecular HBV/HDV entry inhibitors that specifically block myr-preS1_2-48_ lipopeptide binding to NTCP without affecting its bile salt transport function. We analyzed a set of derivatives of the pentacyclic lupane-type triterpenoid betulin, and indeed found individual compounds that were quite potent and selective for myr-preS1_2-48_ lipopeptide binding inhibition and significantly blocked in vitro HDV infection of NTCP-expressing HepG2 cells. These compounds now can be further optimized for increased potency and then can be forwarded for in vivo infection experiments.

## Results

### Screening assay for bile acid transport and myr-preS1_2-48_ lipopeptide binding

**Figure 1:**
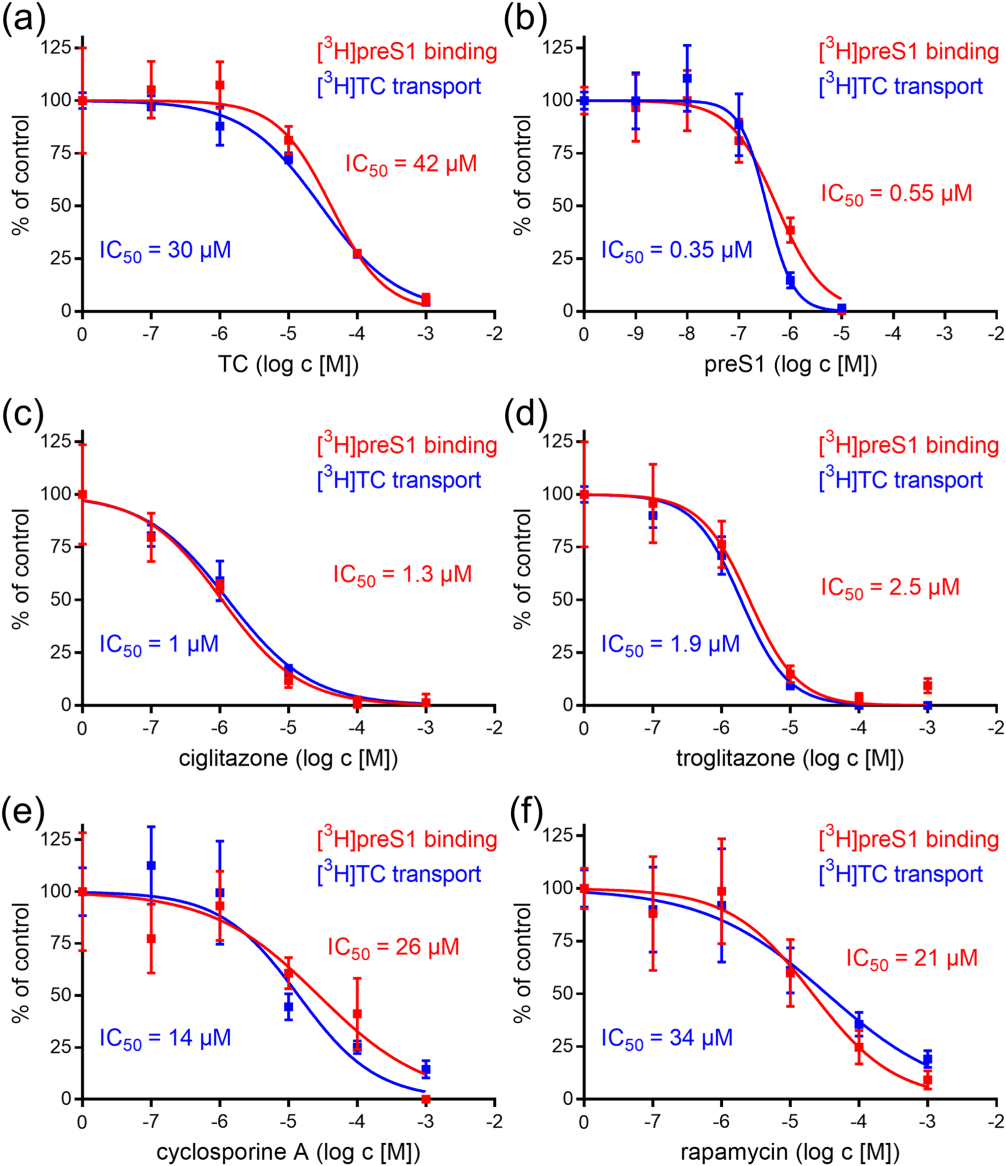
[^3^H]TC transport and [^3^H]preS1 binding screening assay. NTCP-HEK293 cells were seeded onto 96-well plates and were incubated with tetracycline in order to induce expression of the human NTCP protein. Cells without tetracycline treatment were used as 0% controls of [^3^H]TC uptake and [^3^H]preS1 binding, respectively. Cells were subjected to bile acid transport experiments with 1 µM [^3^H]TC and myr-preS1_2-48_ lipopeptide binding experiments with 5 nM [^3^H]preS1, each over 10 min at 37 °C. Experiments were performed with solvent alone (set to 100%) and with increasing concentrations of the indicated inhibitors. The mean of the 0% control was subtracted to calculate net [^3^H]TC transport rates (shown in blue) as well as net [^3^H]preS1 binding rates (shown in red), which are depicted in the diagrams (expressed as % of control at the y-axis). Half maximal inhibitory concentrations (IC_50_) were calculated by nonlinear regression analysis using the equation log(inhibitor) versus response (GraphPad Prism). In order to validate the assay, (**a**) TC and (**b**) myr-preS1_2-48_ lipopeptide were used as inhibitors. Furthermore, the already established NTCP inhibitors (**c**) ciglitazone, (**d**) troglitazone, (**e**) cyclosporine A, and (**f**) rapamycin were analyzed for [^3^H]TC transport inhibition and [^3^H]preS1 binding inhibition. Data represent means ± SD of quadruplicate determinations of representative experiments.

The major goal of the present study was to analyze in parallel the effect of inhibitors on both, bile acid transport via NTCP and myr-preS1_2-48_ lipopeptide binding to NTCP in a cell culture system. This was achieved by plating NTCP-HEK293 cells onto 96-well plates and by using tritium-labelled analytes, being [^3^H]taurocholic acid ([^3^H]TC) and [^3^H]myr-preS1_2-48_ lipopeptide ([^3^H]preS1), which were analyzed in a microplate scintillation counter. For validation of this assay, transport of 1 µM [^3^H]TC first was inhibited by increasing concentrations of TC (concentrations ranging from 0.1 to 1,000 µM) itself and by myr-preS1_2-48_ lipopeptide (concentrations ranging from 0.001 to 10 µM). In addition, binding of 5 nM [^3^H]preS1 was inhibited by myr-preS1_2-48_ lipopeptide itself (concentrations ranging from 0.001 to 10 µM) and by TC (concentrations ranging from 0.1 to 1,000 µM). As shown in Fig. 1a, TC showed nearly equipotent inhibition of the [^3^H]TC transport with IC_50_ of 30 µM and of the [^3^H]preS1 binding with IC_50_ of 42 µM. The same was true for myr-preS1_2-48_ lipopeptide as the inhibitor that revealed IC_50_ of 0.35 µM for [^3^H]TC transport inhibition and IC_50_ of 0.55 µM for [^3^H]preS1 binding inhibition (Fig. 1b). These data clearly confirm the previously shown interference of the transport and receptor function of NTCP^10,18^ and demonstrate the functionality of this screening assay. Furthermore, these data indicate that the myr-preS1_2-48_ lipopeptide has an about 100-fold higher inhibitory potency (IC_50_ = 0.35 µM / 0.55 µM) in both assays compared to the physiological transport substrate TC (IC_50_ = 30 µM / 42 µM). Four reference compounds were analyzed (concentrations ranging from 0.1 to 1,000 µM) which had shown inhibition of the transporter and receptor function of NTCP before, namely ciglitazone^22^, troglitazone^22^, cyclosporine A^23–25^, and rapamycin^26^. Interestingly, all compounds showed comparable inhibition of [^3^H]TC uptake and [^3^H]preS1 binding with IC_50_ values of 1 µM and 1.3 µM for ciglitazone (Fig. 1c), 1.9 µM and 2.5 µM for troglitazone (Fig. 1d), 14 µM and 26 µM for cyclosporine A (Fig. 1e), as well as 34 µM and 21 µM for rapamycin (Fig. 1f), respectively. These compounds therefore can be classified as inhibitors, non-selective for one of the two measured functions of NTCP. Among them, ciglitazone and troglitazone had a tenfold higher inhibitory potency compared to cyclosporine A and rapamycin, demonstrating that diverse compounds differ in their potency as NTCP inhibitors. Cyclosporine A, rapamycin and TC showed comparable inhibitory pattern and potency in both assays, as shown by similar IC_50_ values.

### Screening of betulin derivatives for bile acid transport and [^3^H]preS1 binding inhibition

**Figure 2:**
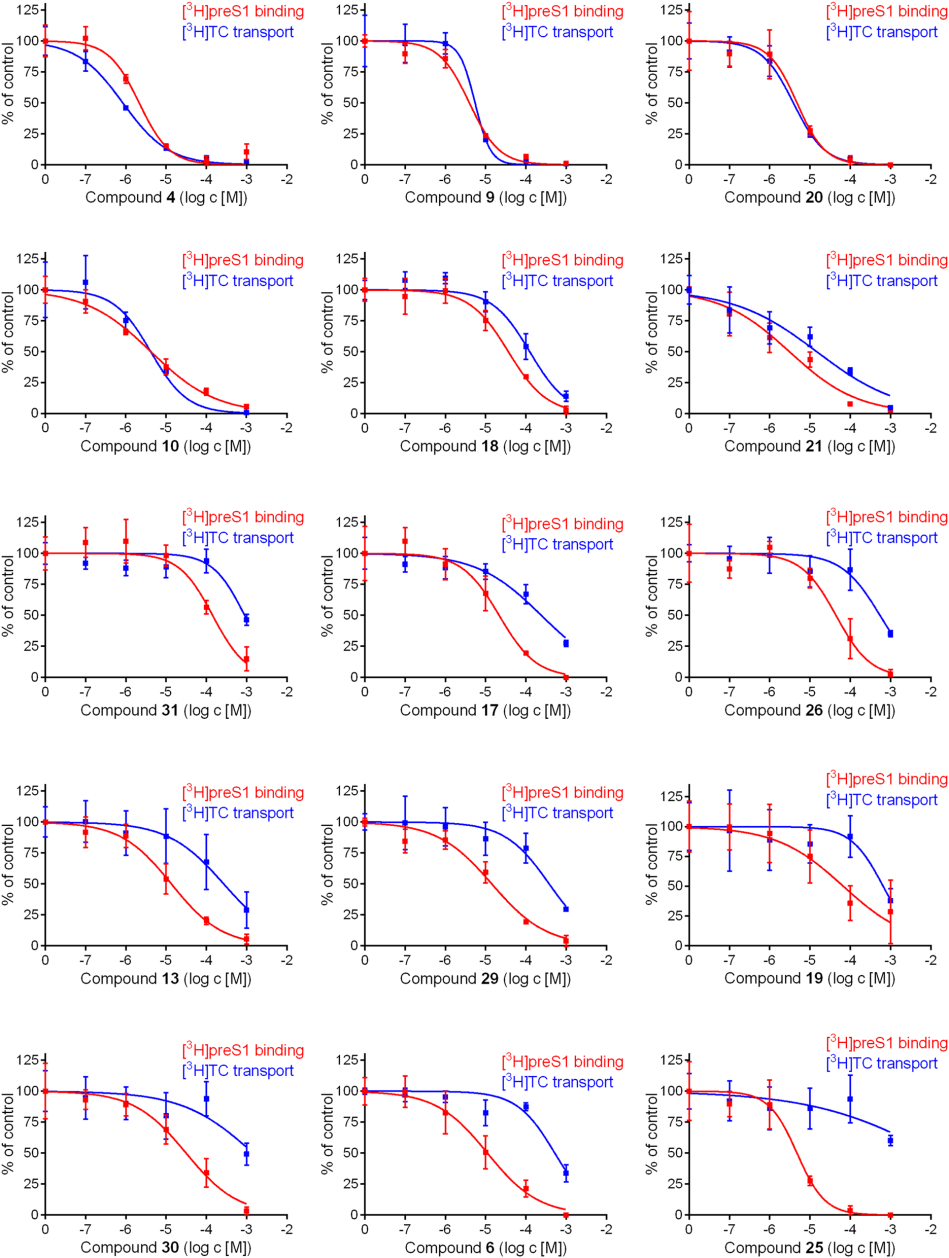
Inhibition of [^3^H]preS1 peptide binding and [^3^H]TC transport by betulin derivatives. NTCP-HEK293 cells were incubated with tetracycline in order to induce NTCP expression and were used for bile acid transport experiments with 1 µM [^3^H]TC and for myr-preS1_2-48_ lipopeptide binding experiments with 5 nM [^3^H]preS1, each over 10 min at 37 °C. Cells without tetracycline treatment were used as 0% controls of [^3^H]TC uptake and [^3^H]preS1 binding, respectively. Experiments were performed with solvent alone (set to 100%) and with increasing concentrations of the indicated inhibitor. The mean of the 0% control was subtracted to calculate net [^3^H]TC transport rates (shown in blue) as well as net [^3^H]preS1 binding rates (shown in red), both expressed as % of control at the y-axis. Half maximal inhibitory concentrations (IC_50_) were calculated by nonlinear regression analysis using the equation log(inhibitor) vs. response (GraphPad Prism). The figure shows selected diagrams. Chemical structures of the compounds are depicted in Table 1, the respective IC_50_ values are listed in Table 2. Data represent means ± SD of quadruplicate determinations (n = 4).

Next, 30 derivatives of betulin were tested in both assays and IC_50_ values were determined for [^3^H]TC transport inhibition and [^3^H]preS1 binding inhibition. Chemical structures of all compounds are depicted in Table 1. All derive from the betulin core structure by chemical modifications. These include methyl, succinyl, acetyl, bromoacetyl, dimethylglutaryl, caffeoyl, oxime, tetrahydropyranyl, cinnamoyl, nicotinoyl, L-aspartyl- or 1,2,4-triazoline-3,5-dione conjugations mostly at positions 3′ and/or 28′, as well as oxidations of the hydroxyl groups at positions 3′ and/or 28′. Compound **29** represents allobetulin and compound **30** 3-oxoallobetulin. All IC_50_ values for [^3^H]TC transport and [^3^H]preS1 binding are listed in Table 2 with their 95% confidence intervals, and selected diagrams are shown in Fig. 2. In the case of [^3^H]TC uptake inhibition, the IC_50_ values ranged from 1 µM (**4**) up to values > 1,000 µM with very low, if any transport inhibition (**3**, **24**, **25, 28** and **30**). This clearly indicates structure–activity relationships over three orders of magnitude. Most of the betulin derivatives showed quite potent inhibition of [^3^H]preS1 binding with IC_50_ values ranging from 2–3 µM (**4**, **8**, **21**) up to 199 µM (**24**), also demonstrating clear structure–activity relationships over a broad inhibitor concentration range.

**Table 1:**
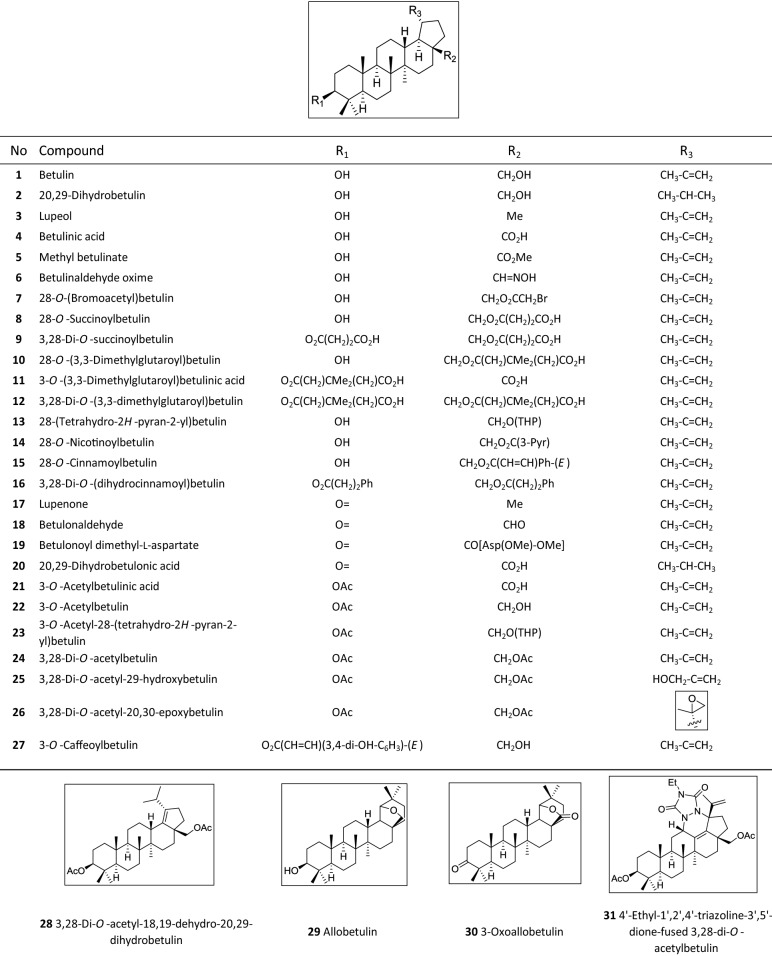
Structures of the betulin derivatives that were used for [^3^H]TC transport inhibition and [^3^H]preS1 binding inhibition experiments. These compounds derived from a TargoSet (Adipogen AG) or were synthesized as outlined elsewhere^52–54^.

Ac, acetyl; Asp, aspartate; Et, ethyl; Me, methyl; Ph, phenyl; Pyr, pyridinyl; THP, 2*H*-tetrahydropyranyl.

**Table 2:** The indicated 31 betulin derivatives were analyzed for [^3^H]TC transport inhibition and [^3^H]preS1 binding inhibition in NTCP-HEK293 cells. IC_50_ values were calculated from a range of five inhibitor concentrations (0.1–1,000 µM) and are listed with their 95% confidence intervals. Selectivity indices for [^3^H]TC transport inhibition/[^3^H]preS1 binding inhibition were calculated from the IC_50_ means. The higher this index, the more selective the respective inhibitor.

Compound	IC_50_ ([^3^H]TC uptake) [µM]^a^	IC_50_ ([^3^H]preS1 binding) [µM]^b^	Selectivity index^c^
Betulinic acid (**4**)	0.65–1.08	1.57–2.93	1
20,29-Dihydrobetulonic acid (**20**)	2.74–5.46	2.95–7.66	1
3,28-Di-*O*-succinoylbetulin (**9**)	3.11–5.02	2.94–10.15	1
28-*O*-(3,3-Dimethylglutaroyl)betulin (**10**)	3.00–5.91	2.48–7.02	1
3-*O*-(3,3-Dimethylglutaroyl)betulinic acid (**11**)	3.08–4.53	3.00–5.21	1
3,28-Di-*O*-(3,3-dimethylglutaroyl)betulin (**12**)	4.17–10.71	4.97–10.14	1
3-*O*-Acetylbetulin (**22**)	36.47–151.80	20.64–87.35	2
Methyl betulinate (**5**)	47.05–99.97	12.66–83.46	2
28-*O*-Succinoylbetulin (**8**)	5.35–10.60	1.61–4.11	3
Betulonaldehyde (**18**)	88.53–175.00	25.98–52.00	3
28-*O*-Nicotinoylbetulin (**14**)	185.40–531.50	36.48–109.10	5
3-*O*-Acetylbetulinic acid (**21**)	7.19–30.33	1.81–5.31	5
3,28-Di-*O*-acetylbetulin (**24**)	> 1,000	52.55–756.00	> 5
3,28-Di-*O*-(dihydrocinnamoyl)betulin (**16**)	176.20–1232.00	24.79–229.20	6
4′-Ethyl-1′,2′,4′-triazoline-3′,5′-dione-fused 3,28-di-*O*-acetylbetulin (**31**)	614.20–1286.00	89.71–224.60	6
Betulin (**1**)	568.80–983.40	48.29–247.10	7
28-*O*-Cinnamoylbetulin (**15**)	72.39–235.50	10.89–31.75	7
3-*O*-Acetyl-28-(tetrahydro-2*H*-pyran-2-yl)betulin (**23**)	119.00–947.20	10.95–113.70	10
Lupenone (**17**)	145.70–396.70	13.10–35.45	11
28-*O*-(Bromoacetyl)betulin (**7**)	63.53–126.00	5.28–11.14	11
3,28-Di-*O*-acetyl-20,30-epoxybetulin (**26**)	331.80–891.20	26.46–72.96	12
3,28-Di-*O*-acetyl-18,19-dehydro-20,29-dihydrobetulin (**28**)	> 1,000	29.95–132.80	> 16
Lupeol (**3**)	> 1,000	26.93–97.94	> 19
28-(Tetrahydro-2*H*-pyran-2-yl)betulin (**13**)	115.80–628.00	9.34–19.54	19
3-*O*-Caffeoylbetulin (**27**)	84.98–195.00	3.35–10.00	22
Allobetulin (**29**)	212.30–674.80	9.67–20.80	27
Betulonoyl dimethyl-L-aspartate (**19**)	297.60–1430.00	21.84–202.60	28
3-Oxoallobetulin (**30**)	> 1,000	17.87–54.43	> 32
Betulinaldehyde oxime (**6**)	359.80–759.00	6.61–18.29	48
20,29-Dihydrobetulin (**2**)	157.80–428.90	1.74–7.14	65
3,28-Di-*O*-acetyl-29-hydroxybetulin (**25**)	> 1,000	5.29–13.04	> 125

^a^ Inhibition of 1 µM [^3^H]TC uptake; ^b^ inhibition of 5 nM [^3^H]preS1 binding; ^c^ calculated from mean IC_50_ TC:preS1.

In addition, ratios between the IC_50_ values for [^3^H]TC transport inhibition and the IC_50_ values for [^3^H]preS1 binding inhibition were calculated and are further referred to as selectivity index. The higher this index, the more selective the respective compound for myr-preS1_2-48_ lipopeptide inhibition. Therefore, the data listed in Table 2 are not only helpful to identify potent myr-preS1_2-48_ lipopeptide binding inhibitors but also to assess their selectivity. These values ranged from an index of 1, including more potent (e.g. **4**) and less potent (e.g. **12**) non-selective inhibitors, up to an index of > 60, including the highly selective and potent inhibitors **2** and **25**. Figure 2 depicts selected diagrams which show the inhibitory curves for the [^3^H]TC transport inhibition (in blue) and [^3^H]preS1 binding inhibition (in red). This panel shows compounds with different characteristics. Within the group of potent [^3^H]preS1 binding inhibitors (IC_50_ < 10 µM), there were (I) non-selective inhibitors (**4**, **9**, **10**, and **20**), (II) poorly selective inhibitors (e.g. **21**), and highly selective inhibitors, being **2** and **25**. Also, from the group of less potent [^3^H]preS1 binding inhibitors (IC_50_ = 11–50 µM), some were poorly selective (e.g. **18**) and others were highly selective (e.g. **13**, **17**, **19** and **30**). Taken together, these data clearly indicate structure–activity relationships of the betulin derivatives for both, bile acid transport inhibition and myr-preS1_2-48_ lipopeptide binding inhibition.

### Most relevant modifications for structure–activity relationships

**Figure 3:**
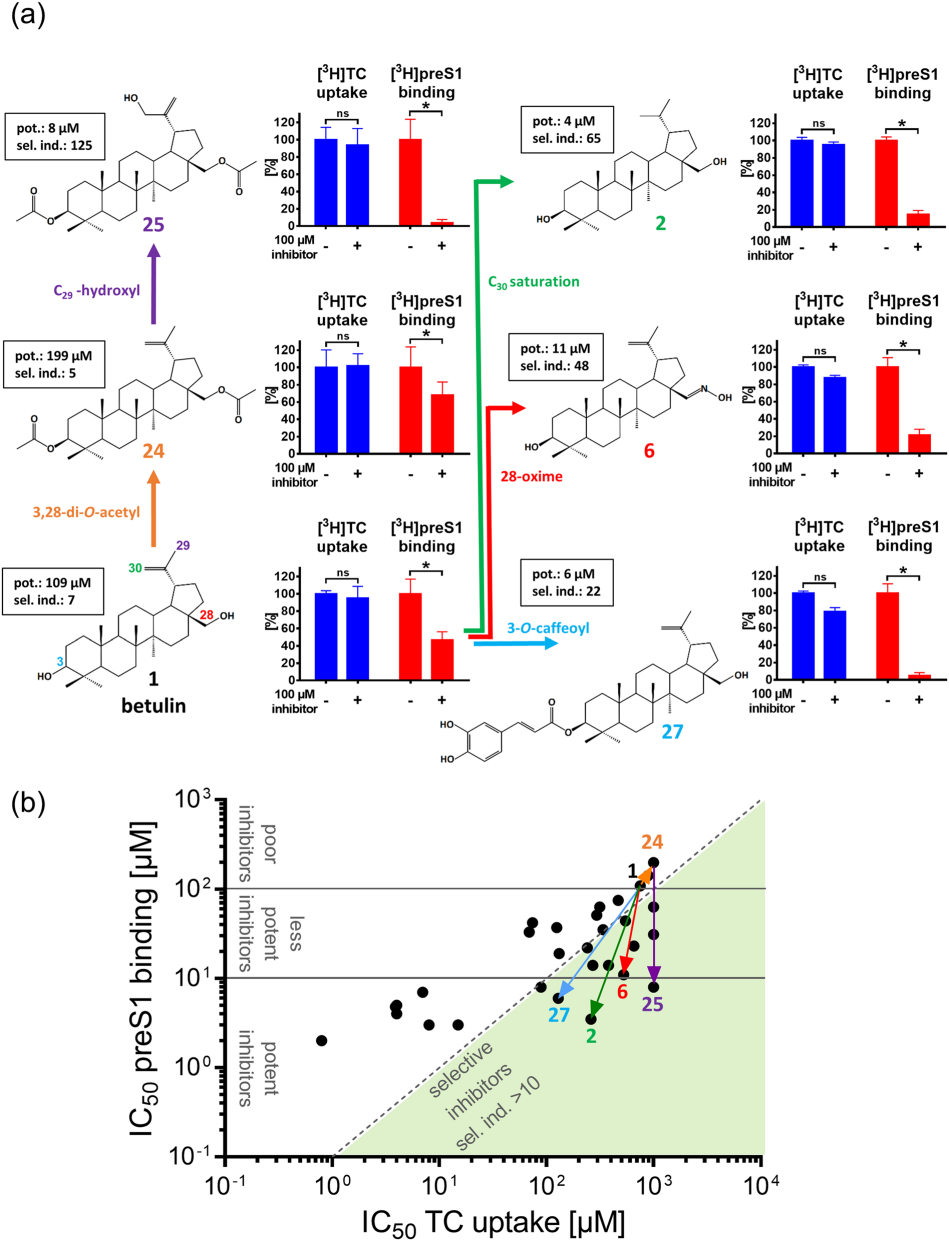
Structure–activity relationships of betulin derivatives for [^3^H]TC transport inhibition and [^3^H]preS1 binding inhibition. Chemical modifications at the lupane skeleton of betulin revealed clear structure–activity relationships for the potency (pot., IC_50_ of [^3^H]preS1 binding inhibition) and virus selectivity index (sel. ind., ratio IC_50_ [^3^H]TC transport inhibition / IC_50_ [^3^H]preS1 binding inhibition). (**a**) 3,28-di-*O*-acetylation (shown in orange), additional C_29_-hydroxylation (shown in purple), 3-*O*-modification with caffeoyl (shown in light-blue), 28-oxime generation (shown in red), and C_30_ saturation (shown in green) of the betulin core structure improve the potency and/or selectivity index of the respective compounds. The respective inhibition pattern are illustrated by bar graphs for [^3^H]TC uptake (shown in dark-blue) and [^3^H]preS1 peptide binding (shown in red), respectively, in the presence or absence of a fix concentration (100 µM) of the respective betulin derivative. Graphs show means ± SD of quadruplicate determinations. *Significantly different with *p* < 0.01 (two-way ANOVA with Sidak's multiple comparison test); ns, not significant. (**b**) The diagram shows all IC_50_ values for [^3^H]TC uptake inhibition and [^3^H]preS1 binding inhibition of the respective compounds. Effects (IC_50_ shifts) of the structural modifications are illustrated by arrows, starting from the core structure betulin (compound **1**). Compounds can be divided into three groups regarding their potency to inhibit [^3^H]preS1 binding: potent inhibitors (IC_50_ ≤ 10 µM), less potent inhibitors (10 µM < IC_50_ < 100 µM) and poor inhibitors (IC_50_ ≥ 100 µM). Compounds with a selectivity index of > 10 are classified as selective inhibitors.

Closer analysis of the structure–activity relationships of the betulin derivatives revealed several interesting patterns that can be used to further optimize the potency and selectivity index of these compounds in order to end up with an even more suitable HBV/HDV entry inhibitor from the class of betulin derivatives. Of note, betulin (**1**) itself showed relatively low inhibitory potency for bile acid transport inhibition (IC_50_ = 748 µM) and [^3^H]preS1 binding inhibition (IC_50_ = 109 µM) resulting in a selectivity index of 7. However, relatively moderate modifications at the betulin core structure revealed significantly improved potency and selectivity index of the individual compounds. Some of these modifications are depicted in more detail in Fig. 3a/3b and involve modifications at positions 3′, 28′, 29′, and 30′. While the diacetylated betulin derivative 3,28-di-*O*-acetylbetulin (**24**) itself has a relatively low potency for [^3^H]preS1 binding inhibition (IC_50_ = 199 µM) and poor selectivity (selectivity index: 5) (Fig. 3, shown in orange), additional C_29_ hydroxylation (**25**) improved the potency for [^3^H]preS1 binding inhibition to IC_50_ = 8 µM and increased the selectivity index to 125 (Fig. 3, shown in purple). 3-*O*-caffeoyl (**27**) conjugation of betulin (**1**) also reduced the IC_50_ value for [^3^H]preS1 binding inhibition to 6 µM and increased the selectivity index to 22 (Fig. 3, shown in light blue). Replacement of the 28-hydroxyl group of betulin by an oxime group (**6**) increased the inhibitory potency for [^3^H]preS1 binding by one order of magnitude (109 µM vs. 11 µM) and also improved the selectivity index (7 vs. 48) (Fig. 3, shown in red). The most successful modification, however, was the saturation of the double bond between C_20_ and C_30_, which increased the potency for [^3^H]preS1 binding inhibition to IC_50_ of 4 µM, while maintaining the low degree of interference with the bile acid transport with IC_50_ of 260 µM (selectivity index: 65) (Fig. 3, shown in green).

### Acidic betulin derivatives are potent, but non-selective inhibitors of NTCP

**Figure 4:**
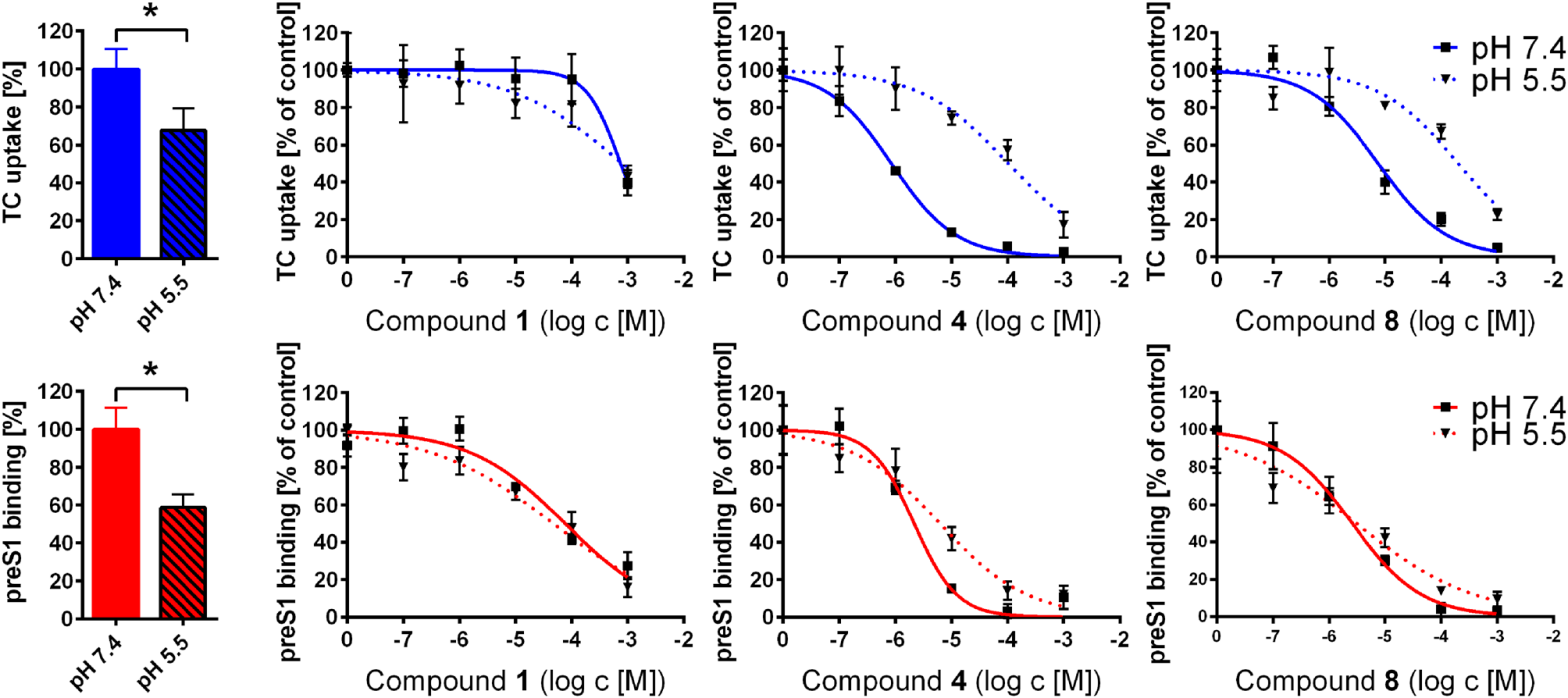
Influence of pH on the inhibition pattern of acidic betulin derivatives. NTCP-HEK293 cells were incubated with tetracycline in order to induce NTCP expression and were used for bile acid transport experiments with 1 µM [^3^H]TC and for myr-preS1_2-48_ lipopeptide binding experiments with 5 nM [^3^H]preS1, each over 10 min at 37 °C. Cells without tetracycline treatment served as control (set to 0%). Experiments were performed with solvent alone (set to 100%) and with increasing concentrations of the indicated inhibitors in the range of 0.1 to 1,000 µM at pH 7.4 and pH 5.5, respectively. Diagrams show inhibition curves at pH 7.4 (solid lines) and pH 5.5 (dashed lines). The mean of the 0% control was subtracted to calculate net [^3^H]TC transport rates (shown in blue) as well as net [^3^H]preS1 binding rates (shown in red), both expressed as % of control at the y-axis. Bar graphs show the relative [^3^H]TC uptake rates (blue bars) and [^3^H]preS1 binding rates (red bars) at pH 7.4 (filled bars) and pH 5.5 (striped bars), respectively, expressed as pmol/10 min/well. Half maximal inhibitory concentrations (IC_50_) were calculated by nonlinear regression analysis using the equation log(inhibitor) vs. response (GraphPad Prism). Data represent means ± SD of quadruplicate determinations (n = 4). *Significantly different with *p* < 0.01 (unpaired t-test with Welch's correction).

All potent (IC_50_ preS1 binding < 10 µM) and non-selective (selectivity index < 10) NTCP inhibitors from the group of betulin derivatives bear at least one acidic group. This applies to compounds **4**, **8—12**, **20**, and **21** (Tab. 1). Based on their pKa values, these compounds are predominantly deprotonated and negatively charged at pH 7.4, similar to bile acids. In order to investigate the role of the pH-dependent negative charge of these compounds, the acidic derivatives **4** (pKa = 4.8) and **8** (pKa = 4.1) were tested for [^3^H]TC uptake inhibition and [^3^H]preS1 binding inhibition at pH 7.4 and pH 5.5. Betulin (**1**) was included as a non-acidic compound for control. In the case of compound **4** a shift of the IC_50_ value for [^3^H]TC uptake inhibition from 0.8 to 103 µM was observed (Fig. 4, upper panel). Compound **8** also showed an IC_50_ shift from 8 to 200 µM by lowering the pH from 7.4 to 5.5. Interestingly, the IC_50_ values for [^3^H]preS1 binding inhibition did not change at lower pH (Fig. 4, lower panel). Apart from this effect on the inhibition pattern, acidification of the incubation medium also affected the absolute [^3^H]TC uptake and [^3^H]preS1 binding rates (Fig. 4, bar graphs). Both functions of NTCP were significantly lower at pH of 5.5, most likely due to pH sensitive amino acid residues at the sites of substrate/preS1 binding.

### Betulin derivatives inhibit HDV infection

**Figure 5:**
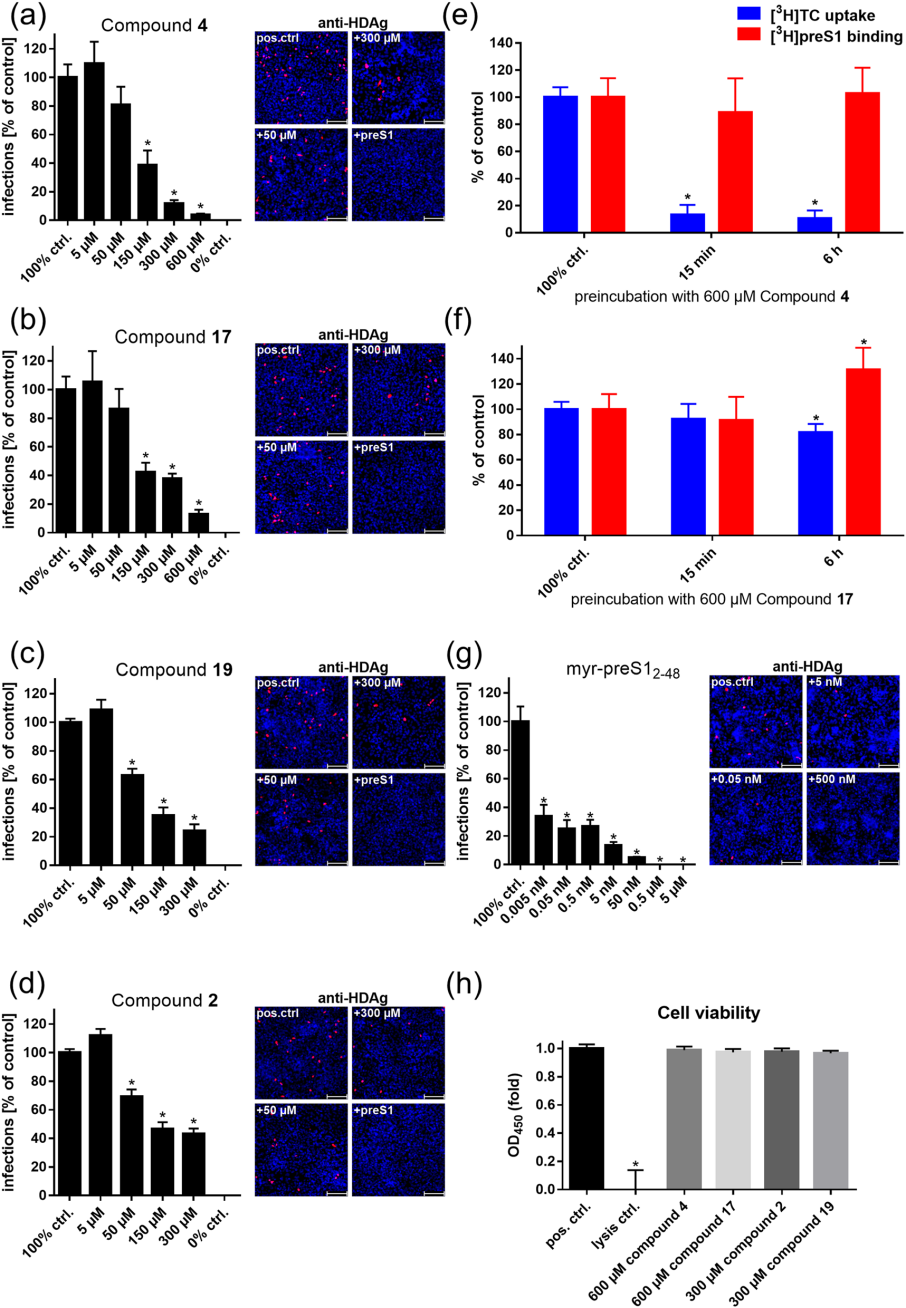
Selected betulin derivatives were used for HDV infection inhibition of NTCP-HepG2 cells. NTCP-HepG2 cells were preincubated for 5 min with the indicated concentrations of (**a**) betulinic acid (compound **4**), (**b**) lupenone (compound **17**), (**c**) betulonoyl dimethyl-L-aspartate (compound **19**), (**d**) 20,29-dihydrobetulin (compound **2**), and (**g**) the myr-preS1_2-48_ peptide in DMEM at 37 °C. Then, cells were additionally inoculated with 700 genome equivalents/cell of HDV particles at 37 °C. After 6 h, cells were washed and further incubated with inhibitor- and virus-free medium, and medium was changed every 3–4 days. At day 9 post infection, cells were fixed and an immunostaining against the HDAg was performed, as a marker of HDV infection. The number of infected cells per well was determined by fluorescence microscopy. NTCP-HepG2 cells incubated without inhibitor were used as control (set to 100% infection rate). Infection experiments in the presence of 0.5 µM myr-preS1_2-48_ peptide served as control for the 0% infection rate. Scale bars: 100 µm. Data represent means ± SEM of three independent experiments each with triplicate determinations (n = 9). (**e**, **f**) Cells were preincubated with 600 µM of compound **4** and compound **17**, respectively, for intervals of 15 min and 6 h at 37 °C. Cells treated with solvent (DMEM) alone served as control (set to 100%) and data from cells without induction of NTCP expression were set to 0%. Subsequently, cells were washed three times with tempered PBS and [^3^H]TC (1 µM) uptake as well as [^3^H]preS1 (5 nM) binding were analyzed, respectively. Means of the 0% controls were subtracted and net [^3^H]TC transport rates and net [^3^H]preS1 binding rates are expressed as % of control at the y-axis. Data were combined from two independent experiments, each with quadruplicate determinations (n = 8). (**h**) Cell viability was determined with an LDH cytotoxicity assay. HepG2 cells treated with HGM w/o test compound were used as positive control and lysed cells as negative control. Data show means ± SD of three independent experiments each with triplicate determinations (n = 9). *Significantly different from control with *p* < 0.01 (two-way ANOVA with Dunnett's multiple comparison test).

Selected betulin derivatives were investigated for their inhibitory potency of in vitro HDV infection of NTCP-expressing HepG2 hepatoma cells. These experiments were of particular interest as in the [^3^H]preS1 assays, [^3^H]preS1 peptide binding is just used as a surrogate parameter for virus binding to NTCP. Of note, these infection experiments were performed in a different cell line as the screening assays and the betulin inhibitors had to be incubated much longer for the in vitro infection experiments (6 h) compared to the [^3^H]preS1 binding assay (15 min). While NTCP-HepG2 cells without inhibitor were used as control (set to 100% infection rate), co-incubation with 0.5 µM of the myr-preS1_2-48_ lipopeptide completely abolished infection of the cells (set to 0% infection rate). As inhibitors for in vitro HDV infection, betulinic acid (**4**) was used as a potent but non-selective inhibitor, 20,29-dihydrobetulin (**2**) as a potent and highly selective inhibitor, as well as lupenone (**17**) and betulonoyl dimethyl-L-aspartate (**19**) as less potent but still selective inhibitors. All four compounds significantly blocked in vitro HDV infection of the NTCP-HepG2 cells in a concentration-dependent manner (Fig. 5), clearly indicating that betulin derivatives represent an interesting novel compound class of HBV/HDV entry inhibitors. Of note, none of the compounds induced toxicity in the cells even after long term incubation at high (300 µM/600 µM) inhibitor concentrations (Fig. 5h).

### Inhibition of HDV infection cannot be attributed to drug-induced internalization of NTCP

Compounds **4** and **17** underwent further investigations to clarify, whether their inhibitory effects on in vitro HDV infection may have been attributed to drug-induced internalization of NTCP. Therefore, NTCP-expressing HepG2 hepatoma cells were pretreated with 600 µM of the respective compound for 15 min or 6 h, respectively (Fig. 5e,f). Subsequently, cells were washed three times with tempered PBS, before [^3^H]TC (1 µM) uptake and [^3^H]preS1 (5 nM) binding were analyzed for 10 min at 37 °C. Cells pretreated with solvent (DMEM) alone served as 100% controls. Data from HepG2 cells without NTCP expression were set to 0%. Even after three intense washing steps, [^3^H]TC uptake was significantly reduced after short (15 min) and long (6 h) term preincubation with compound **4**. In contrast, [^3^H]preS1 binding did not change, clearly confirming plasma membrane localization of NTCP even after long term preincubation with high inhibitor concentrations of 600 µM. In the case of compound **17**, no differences were observed after short term (15 min) preincubation, while [^3^H]TC uptake and [^3^H]preS1 binding slightly changed in opposite directions after long term (6 h) preincubation. These data confirm that also after preincubation with compound **17**, NTCP remained at the plasma membrane.

## Discussion

The identification of the hepatic bile acid transporter NTCP as the high-affinity receptor for HBV and HDV was the basis for the development of a novel class of anti-HBV/anti-HDV drugs, being cell-entry inhibitors targeting NTCP^9^. Since then, many studies have identified compounds that are capable to inhibit the virus receptor function of NTCP: Hepcludex^13^, cyclosporine A and other cyclosporine derivatives^23–25^, ezetimibe^10^, irbesartan^27^, ritonavir^28^, (-)-epigallocatechin-3-gallate^29^, vanitaracin A^30^, Ro41-5253^31^, proanthocyanidin^32^, zafirlukast^33^, sulfasalazine^33^, and Chicago Sky Blue 6B (an azo dye)^33^. Among them, Hepcludex is a peptide-based drug and cannot be administered orally^13^. Furthermore, it has correspondingly complex manufacturing, storage, and distribution requirements. Therefore, it is still desirable to develop a small molecular drug with HBV/HDV cell-entry inhibitor activity that can be orally applied and would be easier to handle.

Many of the other mentioned compounds, e.g. cyclosporine A, were previously known to inhibit the bile acid transport function of NTCP^17^. Others were identified as novel HBV/HDV inhibitors and later revealed their interference with the physiological bile acid transport function of NTCP. Overall, this interference indicates that NTCP domains relevant for substrate binding and transport overlap with the domains mediating myr-preS1_2-48_ lipopeptide / virus binding to NTCP. However, there are also some hints that both functions can be separated. In a previous study, we identified amino acid G158 of the human NTCP as absolutely essential for myr-preS1_2-48_ lipopeptide binding and in vitro HBV and HDV infection. This amino acid is located at a domain (amino acids 157–165) that previously was shown to be involved in myr-preS1_2-48_ binding to human NTCP^9,18^. Accordingly, G158R NTCP mutants were completely insusceptible for in vitro HBV/HDV infection, but still supported transport of bile acids. It was discussed that the larger amino acid side chain of arginine compared to glycine might sterically preclude myr-preS1 peptide binding, while bile acids can still bind to their respective binding pocket^34^. Furthermore, Shimura et al.^25^ showed for the cyclosporine derivative SCY995 higher potency against in vitro HBV infection (IC_50_ < 8 µM) than for bile acid transport inhibition (IC_50_ > 25 µM).

Based on this, the present study was designed to enable the identification of a selective HBV/HDV cell-entry inhibitor from the group of small molecules. Therefore, all test compounds were a priori tested for both, inhibition of [^3^H]preS1 binding to NTCP as a surrogate parameter for HBV/HDV infection and inhibition of [^3^H]TC transport via NTCP. In the present study, a novel chemical class of promising HBV/HDV cell-entry inhibitors was identified, represented by derivatives of the natural pentacyclic lupane-type triterpenoid betulin. Betulin can be isolated from various plant species, belonging to a variety of families such as Betulaceae, Platanaceae, Dilleniaceae, Rhamnaceae, Rosaceae, and Fagaceae^35,36^. Characteristics of the lupane skeleton of betulin are the five-membered E ring and the isopropylidene group^37^. Interestingly, nearly all betulin derivatives tested in the present study significantly inhibited [^3^H]TC transport and [^3^H]preS1 binding. However, the ratios between the IC_50_ values for transport inhibition and for [^3^H]preS1 binding inhibition were quite different and the resulting selectivity indices ranged from 1 up to 65. The latter was the case for compound **2** with an IC_50_ of 4 µM for [^3^H]preS1 binding inhibition and an IC_50_ of 260 µM for bile acid transport inhibition. Some other compounds showed no transport inhibition at all, being **24**, **25**, **28**, and **30**, but at least in the case of **25**, potent inhibition of [^3^H]preS1 peptide binding with IC_50_ of 8 µM. This data clearly indicate structure–activity relationships for both NTCP functions. Of note, only small changes in the molecular structure of the betulin derivatives significantly changed their potency and selectivity index. Moreover, hydroxylation of compound **24** to compound **25**, as an example, improved both factors in parallel: potency for [^3^H]preS1 binding inhibition and the selectivity index. Also other modifications of the betulin molecule (**1**) such as 3-*O*-caffeoly conjugation (compound **27**) or C_20_-C_30_ saturation (compound **2**) significantly improved both factors simultaneously.

Inhibition studies at pH 5.5 revealed additional structure–activity relationships regarding the charge of the tested compounds. All potent but non-selective inhibitors (Fig. 3b) bear at least one acidic group at R1 and/or R2 (see Tab. 1) and show pKa values between 4–5. Therefore, compounds from this group are predominantly deprotonated and negatively charged at physiological pH of 7.4, representing the standard condition in the inhibition assays. For comparison, also conjugated bile acids (pKa = 1–4) are predominantly deprotonated and negatively charged at physiological pH^38–40^. Based on this it can be speculated that acidic betulin derivatives may not only be inhibitors but also transporter substrates of NTCP. This would explain, why these compounds (representatively shown for compounds **4** and **8**) largely lost their [^3^H]TC uptake inhibitory potency when the pH was reduced to 5.5 and, thereby reducing the relative charge of the molecules. In contrast, the [^3^H]preS1 binding inhibitory potency was insensitive to this pH shift and only showed little differences between pH 7.4 and pH 5.5 conditions. This again indicates that the transporter and the receptor function of NTCP can be separated under certain experimental conditions and that blocking of the preS1 peptide binding site of NTCP does not require negative charge of the inhibitor. In contrast to compounds **4** and **8**, betulin itself (compound **1**) does not bear any acidic group. Consequently, betulin could be less active at the bile acid binding site, what would explain the relatively low potency for TC uptake inhibition (IC_50_ = 748 µM) at neutral and acidic pH values. However, no uniform legality could be established for the other chemical modifications of betulin, which would precisely predict the potency and selectivity pattern of the respective betulin derivative. Nevertheless, based on the structure–activity relationships described in the present study it is most likely that even more potent betulin derivatives can be identified, while sparing the cross-reactivity on the bile acid transport function of NTCP.

Among the betulin derivatives, betulinic acid (**4**) particularly underwent intensive toxicological assessment^35,41–43^. In vitro and in vivo model systems consistently categorized betulinic acid (**4**) as a safe compound with low toxicity. More precisely, no toxic effects were observed in rats that obtained single i.p. application of 400 mg/kg or in mice receiving 100 mg/kg i.p. every 3–4 days for a total of six treatments, or 500 mg/kg i.p. once^35,42,43^. Unfortunately, betulinic acid (**4**), although potent in [^3^H]preS1 binding inhibition (IC_50_ = 2 µM), is not selective and showed equipotent inhibition of the bile acid transport function of NTCP at least at physiological pH. Therefore, betulinic acid (**4**) is not regarded as the optimal HBV/HDV entry inhibitor candidate from the betulin class of compounds. Apart from betulinic acid (**4**), betulin (**1**) also has a good safety profile. Betulin (**1**) is approved for medical use in humans and is currently available as a betulin containing gel (Oleogel-S10, Episalvan). It was authorized in 2016 by the European Medicines Agency (EMA) for treatment of partial thickness wounds in adults. This gel contains an extract from birch bark of *Betula pendula* Roth, *Betula pubescens* Ehrh*.,* and hybrids of both species, obtained with *n*-heptane as the extraction solvent. The resulting betulin (**1**) concentrations are in the range of 72–88 mg betulin (**1**) per 100 mg extract. The gel itself is composed of 10% birch bark extract in 90% sunflower oil. Moreover, Episalvan is currently undergoing a phase III efficacy and safety study in patients with inherited epidermolysis bullosa (EB) that started in 2017 with an estimated enrollment of 250 participants (NCT03068780). Based on this good safety profile and the established use in human medicine, betulin (**1**) is an attractive early lead candidate for selective HBV/HDV cell-entry inhibition. However, despite its superior selectivity in comparison to betulinic acid (**4**), the potency for [^3^H]preS1 binding inhibition is relatively low (IC_50_ = 109 µM). Nevertheless, the present study clearly demonstrates that even small modifications of the lupane skeleton of betulin can improve the potency and selectivity of the respective compounds significantly. Therefore, it seems likely that advanced rational drug design can further improve this compound class to end up with a potent and selective HBV/HDV cell-entry inhibitor.

Apart from the above-mentioned clinical application, several previous studies showed that betulin (**1**) and betulin derivatives have a broad spectrum of pharmacological effects, including anti-cancer, anti-viral, anti-microbial, anti-inflammatory, and anti-fibrotic effects^44–48^. Interestingly, betulinic acid (**4**) was also used in HBV infection studies before, which showed inhibition of HBV replication by suppression of manganese superoxide dismutase (SOD2) expression with subsequent mitochondrial reactive oxygen species (ROS) over-generation^49^. Based on this and the results from the present study, betulinic acid (**4**) might have additive HBV/HDV antiviral properties by addressing different cellular targets involved in cell-entry (NTCP) and replication (SOD2). Therefore, further studies with betulin derivatives should take selective NTCP inhibition and potential suppression of SOD2 into account, which was beyond the scope of the present study.

Our [^3^H]preS1 binding assay successfully predicted the inhibitory effect of the betulin derivatives on in vitro HDV infection. A minor but expected limitation is that absolute IC_50_ values varied between both experimental setups. While the qualitative activity pattern of the betulin derivatives remained the same between the two assays, the absolute IC_50_ values for in vitro HDV infection inhibition were about one to two orders of magnitude higher than predicted from the [^3^H]preS1 binding experiments. Most likely, several factors contribute to this effect. One factor could be the different incubation times for [^3^H]preS1 binding (minutes) and in vitro HDV infection experiments (hours). Another factor might be the use of different cell lines and incubation media. Whereas, the [^3^H]preS1 binding assays were performed in NTCP-HEK293 cells in pure DMEM, the infection experiments needed to be performed with NTCP-HepG2 cells in HGM containing significant amounts of bovine serum albumin (BSA) as well as polyethylene glycol (PEG). The latter medium conditions potentially alter free drug concentrations due to protein binding. Lowering of the inhibitory potency of betulinic acid (**4**) due to protein binding has already been identified in a previous study, where this compound was used as inhibitor for the organic anion transporting polypeptide OATP1B3^50^. A further factor could be the different stoichiometry of the single preS1 peptide molecule and the multitude of preS1 domains of the large envelope proteins of HBV/HDV virions. The latter one could need a higher degree of blocked NTCP receptors for successful virus binding and cell-entry inhibition. On this background, it could be beneficial if an NTCP inhibitor would not only be selective, but would also irreversibly block the preS1/virus binding sites at NTCP. However, this was not achieved with the betulin derivatives analyzed in the present study. The data obtained on compounds **4** and **17** more suggest a reversible mode of inhibition for myr-preS1_2-48_ peptide binding to NTCP. Their inhibitory effect on [^3^H]preS1 binding was completely abrogated by washing the cell surface with inhibitor-free medium (see Fig. 5e,f). But, these observations clearly indicate that the inhibitory effects of compounds **4** and **17** on HDV infection cannot be attributed to drug-induced internalization of NTCP.

Of interest are also the long-term effects of compounds **4** and **17** on the transporter and receptor function of NTCP. In the case of compound **4** preincubation with 600 µM significantly and selectively reduced the transporter function of NTCP, while NTCP still was completely preS1 peptide binding competent. As compound **4** (betulinic acid) belongs to the group of acidic betulin derivatives one can speculate that betulinic acid is transported by NTCP, accumulates inside the cell during preincubation and so by *trans*-inhibiton blocks substrate binding but not preS1 peptide binding to the outer surface of the NTCP protein. This again would support the idea that bile acid and preS1 peptide binding to NTCP occur at separate, but overlapping domains. In contrast, compound **17** showed a paradoxical effect after long term preincubation and increased the capacity for [^3^H]preS1 peptide binding to approximately 130%, while the [^3^H]TC uptake rate was slightly reduced. This could be explained by longer lasting allosteric effects of this compound that differently affects the transport and receptor function of NTCP. The mechanism behind these effects could not be clarified in the present study and needs further investigation, including LC–MS/MS supported direct transport experiments with key betulin derivatives.

In conclusion*,* betulin derivatives show clear structure–activity relationships for potent and selective inhibition of the HBV/HDV virus receptor function of NTCP without tackling its physiological bile acid transport function. Therefore, betulin derivatives are promising candidates for further development of HBV/HDV cell-entry inhibitors. Further studies with this compound class should additionally focus on the mode of inhibition (reversible vs. irreversible) and should be extended to all relevant HBV genotypes.

## Methods

### NTCP-expressing cell lines

Human embryonic kidney (HEK293) cells were stably transfected with human NTCP, C-terminally tagged with the FLAG epitope (further referred to as NTCP-HEK293 cells) as reported before^51^. Cells were maintained at 37 °C, 5% CO_2_ and 95% humidity in DMEM/F-12 medium (Thermo Fisher Scientific, Waltham, MA, USA) supplemented with 10% fetal calf serum (Sigma-Aldrich, St. Louis, MO, USA), 4 mM L-glutamine (PAA, Cölbe, Germany) and penicillin/streptomycin (PAA). HepG2 cells stably transfected with NTCP-FLAG (further referred to as NTCP-HepG2^10^) were cultured under the same conditions in DMEM with all supplements listed above, except for L-glutamine. For induction of the transgene, the medium was supplemented with 1 µg/ml tetracycline (Roth, Karlsruhe, Germany) in the case of the NTCP-HEK293 cells or with 2 µg/ml doxycycline (Sigma-Aldrich) in the case of the NTCP-HepG2 cells.

### Inhibitory concentrations (IC_50_) for [^3^H]preS1 binding and [^3^H]TC transport

Bile acid transport measurements were performed in the NTCP-HEK293 cells with [^3^H]TC (20 Ci/mmol, 0.09 mCi/ml, Perkin Elmer, Waltham, USA) as reported before^51^. In parallel, [^3^H]preS1 peptide binding experiments were performed with a tritium-labelled myr-preS1_2-48_ lipopeptide -HBV subgenotype D3- that was purchased from Pharmaron (120 Ci/mmol, 1 mCi/ml, Cardiff, UK). Briefly, cells were seeded onto polylysine-coated 96-well plates, induced with 1 µg tetracycline per ml, and grown to confluence over 72 h at 37 °C. Then, cells were washed once with tempered phosphate buffered saline (PBS, 137 mM NaCl, 2.7 mM KCl, 1.5 mM KH2PO4, 7.3 mM Na2HPO4, pH 7.4) at 37 °C and preincubated with 80 µl DMEM for 5 min at 37 °C. The medium was replaced by 80 µl DMEM containing the respective betulin derivative (concentrations ranging from 0.1 to 1,000 µM) or solvent alone (100% uptake/binding control), and cells were further incubated for 5 min at 37 °C. After this preincubation, bile acid transport experiments were started by adding 20 µl DMEM containing 5 µM [^3^H]TC (final concentration: 1 µM). Binding of [^3^H]preS1 was initiated by adding 20 µl DMEM containing 25 nM [^3^H]preS1 (final concentration: 5 nM). Experiments were stopped after 10 min by two-times washing with ice-cold PBS. For 0% uptake/binding control, the NTCP-HEK293 cells were not induced with tetracycline (-tet). Cell-associated radioactivity of [^3^H]TC or [^3^H]preS1 was quantified by liquid scintillation counting in a Packard Microplate Scintillation Counter TopCount NXT (Packard Instrument Company, Meriden, USA). Transport rates and [^3^H]preS1 binding were determined in counts per minute (cpm). The mean of the 0% control was subtracted and the net [^3^H]TC transport rates and net [^3^H]preS1 binding rates, respectively, were expressed as % of control. Experiments at pH 5.5 were performed the same way, using DMEM acidified with hydrochloric acid (32%, Roth). A set of betulin derivatives was purchased from Adipogen AG (Liestal, Switzerland), including the compounds betulin (**1**), betulinic acid (**4**), lupeol (**3**), betulonaldehyde (**18**), lupenone (**17**), 3-*O*-caffeoylbetulin (**27**), 3,28-di-*O*-acetylbetulin (**24**), 28-*O*-(3,3-dimethylglutaroyl)betulin (**10**), 3,28-di-*O*-(3,3-dimethylglutaroyl)betulin (**12**), 28-*O*-succinoylbetulin (**8**), 3,28-di-*O*-succinoylbetulin (**9**), 3-*O*-(3,3-dimethylglutaroyl)betulinic acid (**11**), and 3-*O*-acetylbetulinic acid (**21**) (TargoSet). All other betulin derivatives were synthesized as outlined elsewhere^52–54^. IC_50_ values were calculated from quadruplicate determinations by GraphPad Prism 6 (GraphPad, San Diego, CA, USA).

### HDV infection experiments

For infection experiments NTCP-HepG2 cells were cultured in 96-well plates in Hepatocyte Growth Medium (HGM) consisting of William’s E Medium (Thermo Fisher Scientific) containing 2% bovine serum albumin (BSA, Roth), 2 mM L-Glutamin (Thermo Fisher Scientific), 100 µg/ml gentamicin (Thermo Fisher Scientific), 10 nM dexamethasone (Sigma-Aldrich), 1 mM sodium pyruvate (Thermo Fisher Scientific), 1 × Insulin-Transferrin-Selen (Thermo Fisher Scientific), 2% DMSO (Merck, Darmstadt, Germany), 4% polyethylene glycol (Sigma-Aldrich) and 2 µg/ml doxycycline (Sigma-Aldrich) as reported^10^. Subsequently, cells were washed with DMEM and cultured in HGM supplemented with 2% DMSO, 2% BSA and 2 µg/ml doxycycline. NTCP-HepG2 cells were preincubated for 5 min with inhibitors solved in 80 µl HGM per well in concentrations ranging from 5 µM to 300 µM (compounds **2** and **19**) or from 5 µM to 600 µM (compounds **4** and **17**). HDV stock solved in 20 µl HGM per well was added for infection and cells were incubated for 6 h with final concentration of 700 genome equivalents/cell of HDV particles. HDV production was done in vitro as described before^55,56^. RT-qPCR was performed to determine genome equivalents. Every three days medium was changed until cells were fixed at 9 days post infection with 3% paraformaldehyde (Sigma-Aldrich) in PBS, for 30 min at room temperature (RT). Cells were permeabilized with 0.2% Triton X 100 (Roth) in PBS for 30 min at RT, and blocked by incubation with 5% bovine serum albumin (Roth) in PBS, for 30 min at RT. Then, cells were immunostained with purified human anti-HDV-positive serum at 37 °C for 1 h (1:400 dilution). Goat anti-human IgG secondary antibody coupled to Alexa Fluor fluorophore (1:400 dilution, Thermo Fisher Scientific) was added for 1 h at 37 °C for detection of Hepatitis Delta antigen (HDAg) as described before^34^. Nuclei were stained with Hoechst 33342 (1 µg/ml, Thermo Fisher Scientific).

### Preincubation studies in NTCP-HepG2 cells

NTCP-expressing HepG2 hepatoma cells were seeded onto rat tail collagen coated 24-well plates, induced with 2 µg doxycycline per ml, and grown to confluence over 72 h at 37 °C. For experiments, cells were preincubated with a fix concentration (600 µM) of compound **4** and compound **17**, respectively, solved in 300 µl DMEM for intervals of 15 min or 6 h at 37 °C. Cells treated with solvent alone (DMEM) were used as 100% controls. Cells without induction of NTCP expression by doxycycline were used as 0% controls. Subsequently, cells were washed three times with tempered PBS. Then, [^3^H]TC uptake and [^3^H]preS1 binding experiments were started by adding 300 µl of 1 µM [^3^H]TC or 5 nM [^3^H]preS1, respectively. After 10 min, experiments were stopped by washing the cells 5 times with ice-cold PBS. Cell-associated radioactivity was quantified by liquid scintillation counting as described above. [^3^H]TC uptake and [^3^H]preS1 binding rates were determined in pmol/mg protein/10 min. Means of the 0% controls were subtracted and the net [^3^H]TC uptake and net [^3^H]preS1 binding rates, respectively, were expressed as % of control. Data were calculated from two independent experiments, each with quadruplicate determinations by GraphPad Prism 6 (GraphPad, San Diego, CA, USA).

### Cytotoxicity assay

Pierce lactate dehydrogenase (LDH) cytotoxicity assay (Thermo Fisher Scientific) was performed according to the manufacturer’s protocol. Briefly, NTCP-HepG2 cells were incubated with 300 µM or 600 µM of the respective betulin derivatives over 6 h at 37 °C. 45 min before the end of incubation, lysis buffer was added to the maximal LDH control. After 6 h, 50 µl of each sample and control medium were transferred to a new well plate, 50 µl of the reaction mixture were added and incubation was performed over 30 min at room temperature. Finally, the reaction was stopped by adding 50 µl of stop solution and samples were measured by ELISA reader (GloMax-Multi Detection System, Promega, Madison, WI, USA).

### Structure modeling and pKa determination

2D compound structures were generated using the MAESTRO 12.2 Molecular Modeling Interface of SCHRÖDINGER, Inc. (New York City, NY, USA). The Jaguar pKa prediction module of the SCHRÖDINGER software was used to calculate pKa values of compounds **4** and **8**.

### Statistics

Determination of IC_50_ values was done by nonlinear regression analysis using the equation log(inhibitor) vs. response settings of the GraphPad Prism 6.0 software (GraphPad). Data of bile acid transport and [^3^H]preS1 binding are expressed as means ± SD from quadruplicate determinations. IC_50_ values are listed with their 95% confidence intervals in Table 2. In addition, mean IC_50_ values were calculated for each experiment. Infection studies show data from three independent experiments, each with triplicate determinations represented as means ± SEM. Statistical analyses of the HDV infection experiments and NTCP-HepG2 preincubation studies were performed by two-way ANOVA, followed by Dunnett's multiple comparison test by GraphPad Prism 6.0, considering *p* < 0.01 as statistically significant.

## Data Availability

Obtained and analyzed data of this study are available from the corresponding author on request.
